# High drug-loading gold nanoclusters for responsive glucose control in type 1 diabetes

**DOI:** 10.1186/s12951-019-0505-z

**Published:** 2019-06-03

**Authors:** Yujie Zhang, Mingxin Wu, Wubin Dai, Min Chen, Zhaoyang Guo, Xin Wang, Di Tan, Kui Shi, Longjian Xue, Sheng Liu, Yifeng Lei

**Affiliations:** 1 School of Power and Mechanical Engineering & The Institute of Technological Sciences, Wuhan University, Wuhan, 430072 China; 2 School of Material Science and Engineering, Wuhan Institute of Technology, Wuhan, 430205 China; 3 Department of Internal Medicine & Geriatrics, Wuhan University Zhongnan Hospital, Wuhan, 430071 China

**Keywords:** Diabetes, Glucose-responsive, Gold nanoclusters, Drug release

## Abstract

**Background:**

Diabetes is one of the biggest medical challenges worldwide. The key to efficiently treat type 1 diabetes is to accurately inject insulin according to the blood glucose levels. In this study, we aimed to develop an intelligent insulin-releasing gold nanocluster system that responds to environmental glucose concentrations.

**Results:**

We employed gold nanoclusters (AuNCs) as a novel carrier nanomaterial by taking advantage of their high drug-loading capacity. We prepared AuNCs in the protection of bovine serum albumin, and we decorated AuNCs with 3-aminophenylboronic acid (PBA) as a glucose-responsive factor. Then we grafted insulin onto the surface to obtain the glucose-responsive insulin-releasing system, AuNC-PBA-Ins complex. The AuNC-PBA-Ins complex exhibited high sensitivity to glucose concentration, and rapidly released insulin in high glucose concentration in vitro. In the type 1 diabetic mouse model in vivo, the AuNC-PBA-Ins complex effectively released insulin and regulated blood glucose level in the normoglycemic state for up to 3 days.

**Conclusions:**

We successfully developed a phenylboronic acid-functionalized gold nanocluster system (AuNC-PBA-Ins) for responsive insulin release and glucose regulation in type 1 diabetes. This nanocluster system mimics the function of blood glucose regulation of pancreas in the body and may have potential applications in the theranostics of diabetes.

**Electronic supplementary material:**

The online version of this article (10.1186/s12951-019-0505-z) contains supplementary material, which is available to authorized users.

## Background

Diabetes is one of the most common chronic diseases worldwide and has become a third treat to public health, after cardiovascular diseases and cancers [1]. In 2017, diabetes affected 451 million adult patients worldwide, causing considerable burden for the affected individuals and society [1]. The hallmark of diabetes is the high levels of blood glucose in the body (hyperglycemia), which occurs because the body can not produce enough insulin (type 1 diabetes) or use insulin effectively (type 2 diabetes) [2]. Persistent hyperglycemia leads to complications affecting various organs within the body, such as the heart, eyes, kidneys and nerves [3].

The primary goal of type 1 diabetes treatment is to attain normal glucose levels (normoglycemia) in the body. Intensive insulin therapy is considered to be the standard treatment for type 1 diabetes. Traditionally, patients carry out blood glucose testing by finger pricking and receive insulin injection to maintain normoglycemia [3]. However, traditional methods not only bring physiological pain and inconvenience to the patients, but also cause a high risk of complications due to the inaccurate injection of insulin [3]. A novel strategy of closed-loop insulin delivery has recently been developed for diabetes treatment [4–7], with products such as Medtronic MiniMed^®^ 670G. This strategy combines subcutaneous continuous glucose monitoring system [8], algorithms, and intraperitoneal insulin pump [9–11]. However, closed-loop insulin delivery is limited in application because of its high cost and technical sophistication [8].

Thus, the development of an intelligent insulin delivery system that responds to environmental glucose concentrations is urgently needed [12]. In this study, we aim to develop a glucose-responsive insulin-releasing system based on gold nanoclusters for glucose control in type 1 diabetes. Stimuli-responsive materials for drug delivery have attracted extensive interest in recent years [13, 14], as these materials can be designed to control drug delivery in response to specific stimuli, such as pH, enzyme concentration or redox gradients [13, 14]. Typically, glucose-responsive insulin-releasing materials have been designed based on glucose-sensing elements of glucose oxidase, glucose binding protein, or phenylboronic acid (PBA) [15, 16]. Among them, a commonly used strategy to design glucose-responsive materials takes advantages of the ability of PBA and its derivatives to combine reversibly with *cis*-diol units [17–20].

Moreover, nanocarriers have been widely used in drug delivery because of their unique properties, such as tunable size and surface properties, and multiple functional capabilities [15, 21]. Glucose responsiveness can be achieved with mesoporous silica nanoparticles [17], hydrogel-based nano-networks [22], and enzyme nanocapsules [23]. Among various nanomaterials, gold nanoclusters (AuNCs) are a novel gold-based nanomaterial that has attracted special attention because of their ultra-small-size effect, good biocompatibility, surface chemistry, and extremely high drug-loading capacity [24–26].

In this study, we aimed to develop an AuNC-based glucose-responsive insulin-releasing system for glucose control in type 1 diabetes. We prepared AuNCs with the protection of bovine serum albumin (BSA) and decorated the AuNCs with PBA molecule as a responsive factor. Insulin was grafted on the surface to construct the glucose-responsive insulin-releasing system, AuNC-PBA-Ins complex. This nanocomplex increased the efficiency of insulin release in response to glucose concentration, and regulated the blood glucose levels of type 1 diabetic mice in the normoglycemic range for up to 3 days, mimicking the pancreatic function of blood glucose regulation in the body.

## Materials and methods

### Materials

All chemical reagents were commercially available and used without further purification. Chloroauric acid (HAuCl_4_·3H_2_O), glycine, ninhydrin hydrate, d-glucose, sodium acetate, and sodium bicarbonate were purchased from Sigma-Aldrich. BSA, 3-aminophenylboronic acid (PBA-NH_2_), 1-ethyl-(3-dimethylaminopropyl)-carbodiimide hydrochloride (EDC), *N*-hydroxysuccinimide (NHS), morpholine ethanesulfonic acid (MES), and glutaraldehyde solution were obtained from Aladdin. Bovine insulin was purchased from Shanghai Yuanye Bio-Technology. Ultrapure water (Milli-Q) with a resistivity of 18.2 MΩ was used as a general solvent throughout the study.

### Preparation of gold nanoclusters (AuNCs)

**Fig. 1:**
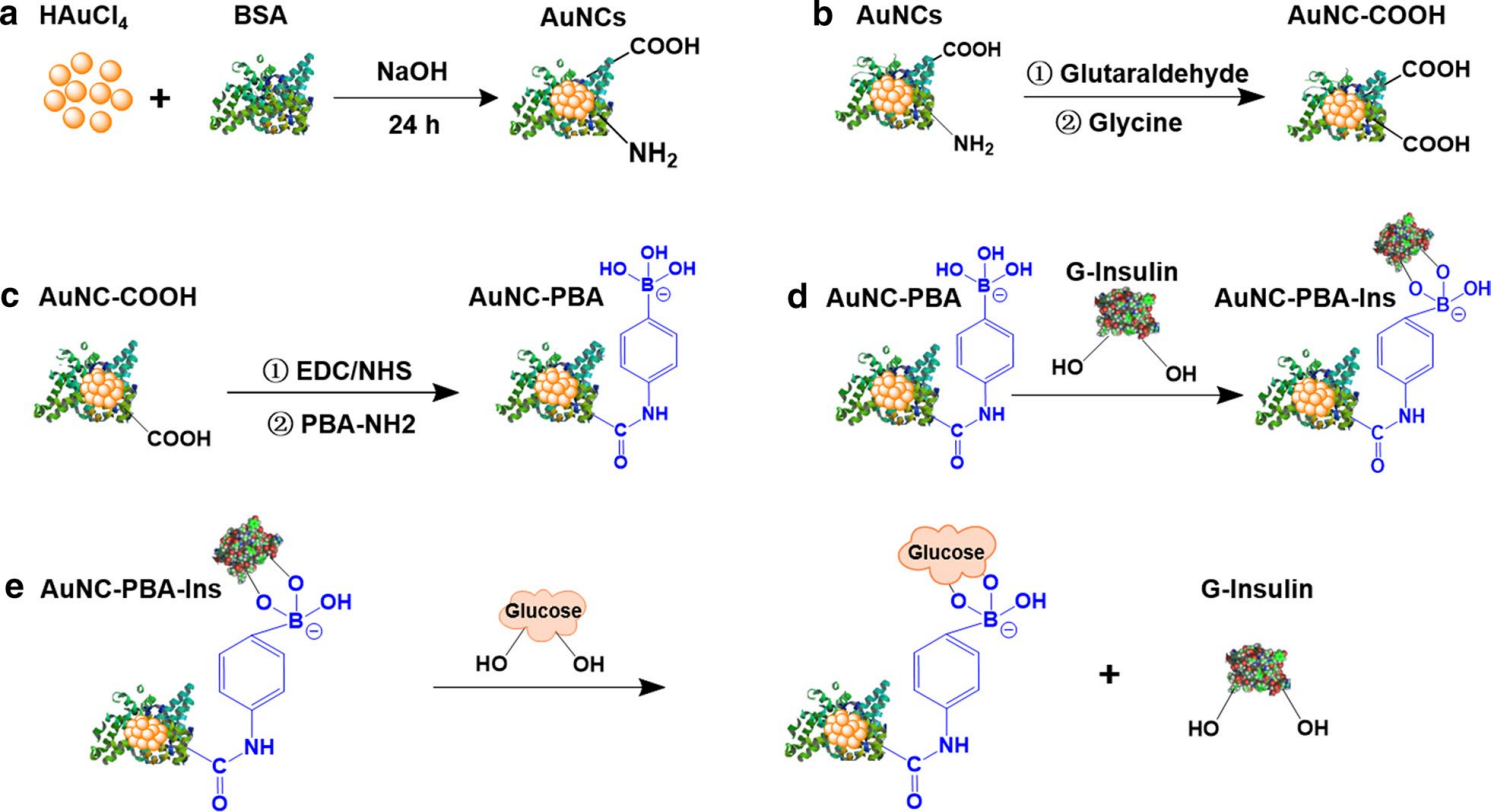
Schematics of synthesis and responsive insulin release of gold nanoclusters (AuNCs). **a** Synthesis of AuNCs. **b** Conversion of amino group into carboxyl group on the surface of AuNCs. **c** Grafting of phenylboronic acid (PBA) molecule onto AuNCs to obtain AuNC-PBA. **d** Grafting of G-Insulin onto AuNC-PBA to achieve glucose-responsive insulin-releasing AuNC-PBA-Ins complex. **e** Mechanism of insulin release from AuNC-PBA-Ins for glucose regulation

**Fig. 2:**
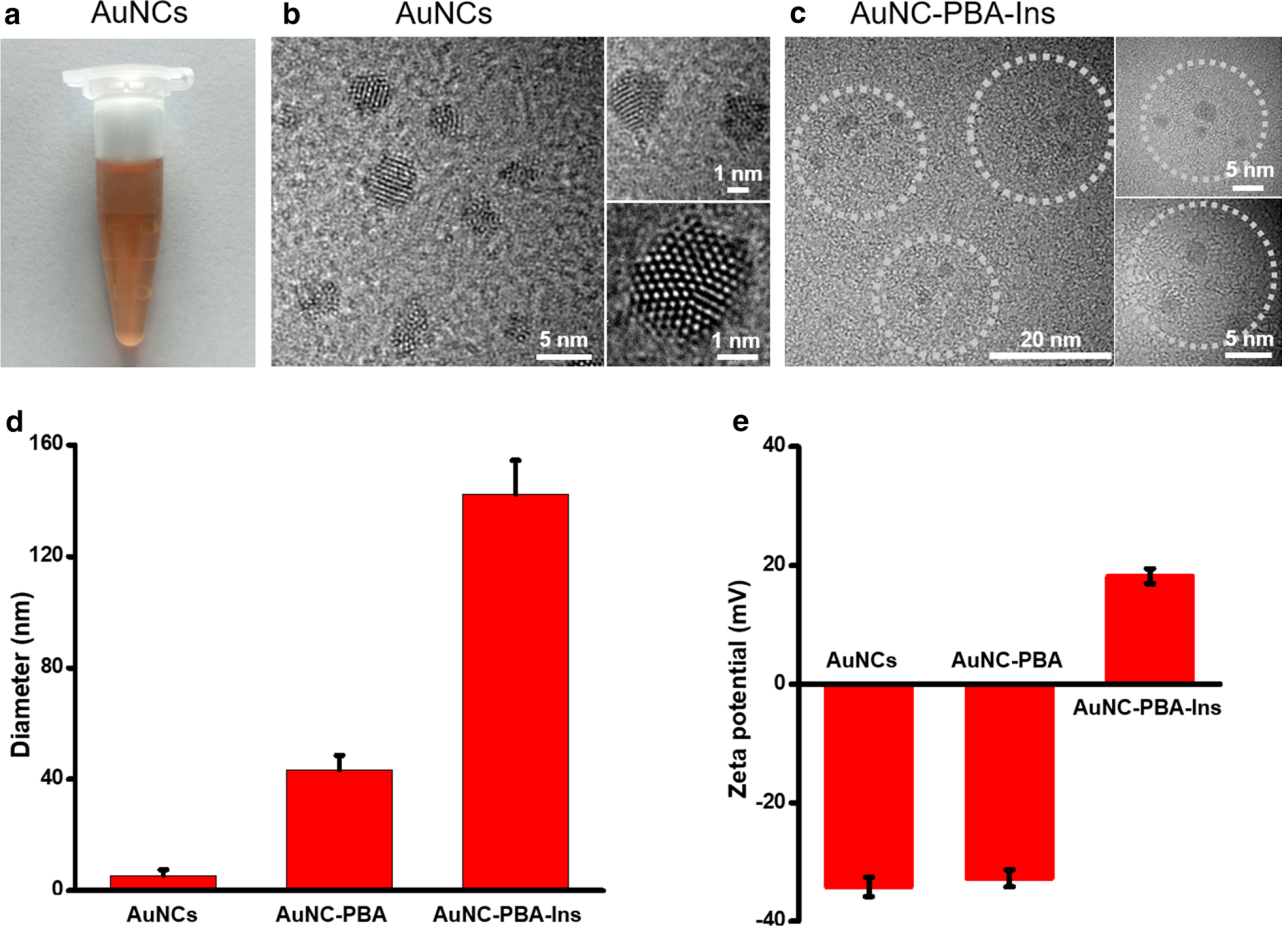
Characterization of gold nanoclusters. **a** The appearance of AuNC solution. **b** TEM images of AuNCs. **c** TEM image of AuNC-PBA-Ins complex. **d** Diameter of AuNCs during synthesis measured by DLS measurement. **e** Surface charge of AuNCs during synthesis

AuNCs were prepared with the protection of BSA according to a previously reported method [27]. In brief, 25 mL of 10 mM HAuCl_4_ solution was added to 25 mL of 50 mg/mL BSA solution under vigorous stirring, and 1 M NaOH solution was introduced to ensure a pH of 12 for the reaction (Fig. 1a). The mixture was incubated at 37 °C for 24 h with protection from light to form AuNCs (Fig. 1a). The color of the solution gradually changed from light yellow to light brown and then to deep brown (Fig. 2a). Excess chemicals were washed twice and removed through ultracentrifugation with a molecular weight cutoff tube (MWCO: 3000, Millipore) at 6000 rpm for 20 min.

The concentration of amino groups on the surface of the prepared AuNCs was determined by ninhydrin colorimetric test, a method used for quantification of the amino groups of amino acids [28]. In brief, purple color was developed on heating the amino groups of the samples with ninhydrin, and the color of the solution was proportional to the concentration of amino groups. Then the amino groups on the samples were quantified using a microplate reader at 570 nm (Thermo Scientific Multiskan FC).

### Removal of amino groups on AuNCs

The amino groups on the surface of AuNCs were converted into carboxyl groups with glutaraldehyde and glycine (Fig. 1b, Additional file 1: Figure S1), according to previous descriptions [29]. In brief, 3.5% (v/v) glutaraldehyde was introduced into the AuNC solution (Additional file 1: Figure S1a), and reacted for 4 h at room temperature. The product was washed twice by ultracentrifugation with a molecular weight cutoff tube (MWCO: 10000) at 6000 rpm for 20 min. The extra aldehyde groups on the surface of the AuNCs were further removed by treatment with 10 mM glycine/10 mM sodium citrate for 30 min at room temperature (Additional file 1: Figure S1b). The products were washed twice by ultracentrifugation. Finally, we obtained AuNCs having only carboxyl groups on the surface, labeled as AuNC-COOH (Fig. 1b).

### Functionalization of AuNC-COOH with PBA

The carboxyl groups on AuNC-COOH (7.5 mM, 2 mL) were activated by incubation in a solution of EDC (20 mM) + NHS (10 mM) in MES buffer (10 mM, 2 mL) for 15 min at room temperature (Fig. 1c①) [30]. The activated AuNC-COOH was ready to react with the amino group on PBA-NH_2_ (Fig. 1c②). With the reaction between the amino group and activated carboxyl group, we immobilized the PBA-NH_2_ molecule (4 mM, 4 mL) onto the surface of AuNC-COOH for 15 h at room temperature (Fig. 1c②). The product (AuNC-PBA) was ultracentrifuged twice at 6000 rpm for 20 min (MWCO: 3000) to remove the excess chemicals.

### Grafting of G-Insulin onto AuNC-PBA

Gluconic acid modified bovine insulin (G-Insulin) was prepared according to previous publications (Additional file 1: Figures S2, S3) [31]. We grafted G-Insulin onto AuNC-PBA by adding G-Insulin (0.5 mM, 1 mL) into AuNC-PBA (2 mM, 5 mL). After 24 h of reaction at room temperature, the obtained product was labeled as AuNC-PBA-Ins (Fig. 1d). The physically adsorbed G-Insulin on the surface of the AuNC-PBA-Ins was washed twice and removed by ultracentrifugation with a molecular weight cutoff tube (MWCO: 10000) at 6000 rpm for 20 min.

The loading of G-Insulin on the nanocomplex was calculated by subtracting the amount of G-Insulin remaining in the washing buffer from the amount of G-Insulin initially introduced into the reaction.

### Characterization

We used dynamic light scattering (DLS, Malvern Nano ZS) to measure the diameter, polydispersity index (PDI), and surface charge of the prepared AuNCs. We examined the morphology of the AuNCs by transmission electron microscopy (TEM, Tecnai G2 F30). We performed Fourier-transform infrared spectroscopy (FTIR, Bruker, V70) and X-ray photoelectron spectroscopy (XPS, Thermo Fisher, Escalab 250Xi) to analyze the chemical structures and elemental composition of the samples, respectively. We measured the gold concentration in the AuNC-PBA-Ins solution with inductively coupled plasma mass spectrometry (ICP-MS; Agilent 7700), and we evaluated the conformational structure of insulin with circular dichroism (CD) spectrometer (BioLogic MOS-500).

### In vitro insulin release

We prepared glucose solution at concentrations of 100 mg/dL (5.55 mM), 200 mg/dL (11.11 mM), and 400 mg/dL (22.22 mM). We added the AuNC-PBA-Ins complex (with insulin at a concentration of 0.79 mM, 30 μL) into the above glucose solutions (1.5 mL) and incubated at 37 °C. After different time points, we centrifuged the sample at 10,000 rpm for 2 min, and we collected 5 μL of the supernatant for insulin analysis. We determined the released insulin concentration in solution using Bradford protein assay kit (Beyotime). We draw the accumulative insulin release from AuNC-PBA-Ins complex as a function of incubation time.

### In vivo studies

#### Animals

We obtained C57B6 mice (8-week-old, male) from the Vital River Laboratory Animal Center (Beijing, China), and raised them under supervision in a specific pathogen free (SPF) environment. All animal studies were approved by the Institutional Animal Care and Use Committee (IACUC) at Wuhan University (AUP number: #S01317091A).

#### Induction of type 1 diabetes in experimental mice

We intraperitoneally injected streptozocin (STZ, Sigma Aldrich) into the mice at 100 mg/kg once per day for 5 days (Additional file 1: Figure S4a). We collected blood (~ 5 μL) daily from the tail vein of the mice for glucose detection with Sinocare GA-3 glucose monitor (Sannuo, China). Type 1 diabetes was successfully induced in the mice when the blood glucose level surpassed 16 mM, and the mice were ready for experimental use (Additional file 1: Figure S4a, b).

#### Glucose regulation in type 1 diabetic mice

We evaluated the efficacy of glucose regulation in the STZ-induced type 1 diabetic mice. We divided the mice into three groups for subcutaneous administration with saline, insulin, and AuNC-PBA-Ins solution (n = 5–6 per group). We injected 100 μL of each aqueous solution into the subcutaneous dorsum of the mice (insulin dose of 10 μmol/kg body weight) and monitored the blood glucose levels over time.

#### In vivo insulin detection

To measure the insulin concentration in vivo, we collected blood samples (~ 20 μL) from the tail vein of the mice into microtubes precoated with anticoagulants of sodium citrate and preserved the serum samples at − 20 °C. We used a bovine insulin enzyme-linked immunosorbent assay (ELISA) kit (Shanghai Yuanye Bio-Technology) to determine the plasma bovine insulin concentrations over time.

#### Glycated albumin (GA) level in mice

To evaluate the GA level in mice, we analyzed the collected serum samples at 1 day before and at 10 days after drug administration, using a mouse GA kit (Shanghai Yuanye Bio-Technology) and mouse albumin kit (Shanghai Yuanye Bio-Technology). We calculated the GA level in mice by measuring the ratio of GA concentration over total albumin concentration in the serum.

### Statistical analysis

Data were presented as the mean ± standard deviation (s.d.). The in vitro experiments were performed in three independent experiments with at least three repetitions for each condition. The in vivo experiments were performed with 5–6 mice in each group. Statistical analysis of the samples was performed using Student’s *t*-test, and a *p* value of less than 0.05 was considered statistically significant.

## Results and discussion

### Synthesis and characterization of AuNCs and AuNC-PBA-Ins

We synthesized BSA-protected AuNCs via progressive reduction of Au^3+^ in BSA (Fig. 1a). The obtained AuNCs had a deep-brown appearance in solution (Fig. 2a), a well-defined core structure (Fig. 2b) with a diameter of 1.7 ± 0.2 nm identified by TEM measurement (Fig. 2b, Additional file 1: Table S1), and a hydrodynamic diameter of 5.4 ± 2.0 nm measured by DLS measurement (Fig. 2d, Additional file 1: Table S1). The AuNCs had a negative charge of − 34.2 ± 1.6 mV (Fig. 2e), as the pH of the reaction (12) was higher than the pKa of BSA (5.1) [27]. The negatively charged surface of AuNCs indicated that the AuNC solution had good stability for storage and application.

We then converted the amino groups on the AuNCs into carboxyl groups to obtain AuNC-COOH (Fig. 1b, Additional file 1: Figure S1). The surface free amino groups were reduced from 33.75 mM on AuNCs into 0.15 mM on the surface of AuNC-COOH (Table 1), revealing the successful conversion of the amino groups into carboxyl groups (Fig. 1b).

**Table 1:** Concentration of amino groups on gold nanoclusters

Samples	Amino group (mM)
AuNCs	33.75 ± 7.08
AuNC-COOH	0.15 ± 0.06

We grafted the PBA-NH_2_ molecule onto the activated AuNC-COOH to obtain AuNC-PBA (Fig. 1c). The AuNC-PBA had a hydrodynamic diameter of 43.3 ± 5.2 nm (Fig. 2d, Additional file 1: Table S1) and a surface charge of − 32.7 ± 1.4 mV (Fig. 2e, Additional file 1: Table S1) due to the negative charge from boronic acid in solution [13].

We then grafted G-Insulin onto AuNC-PBA through covalent bonding between the charged form of PBA and the vicinal diols of G-Insulin to obtain the glucose responsive releasing AuNC-PBA-Ins complex (Fig. 1d). The gluconic acid modification of bovine insulin (G-Insulin) did not affect the bioactivity of native insulin (Additional file 1: Figures S2, S3). The AuNC-PBA-Ins complex had a diameter of 22.6 ± 2.0 nm, as revealed by TEM observation (Fig. 1c, Additional file 1: Table S1), and contained between 3 and 8 AuNCs (Fig. 1c). The AuNC-PBA-Ins complex had a hydrodynamic diameter of 142.4 ± 11.9 nm, as demonstrated by DLS (Fig. 1d, Additional file 1: Table S1), with a surface charge of 18.2 ± 1.2 mV (Fig. 2e, Additional file 1: Table S1), and remained moderately dispersed in solution with a PDI of 0.314 ± 0.065 (Additional file 1: Table S1).

**Fig. 3:**
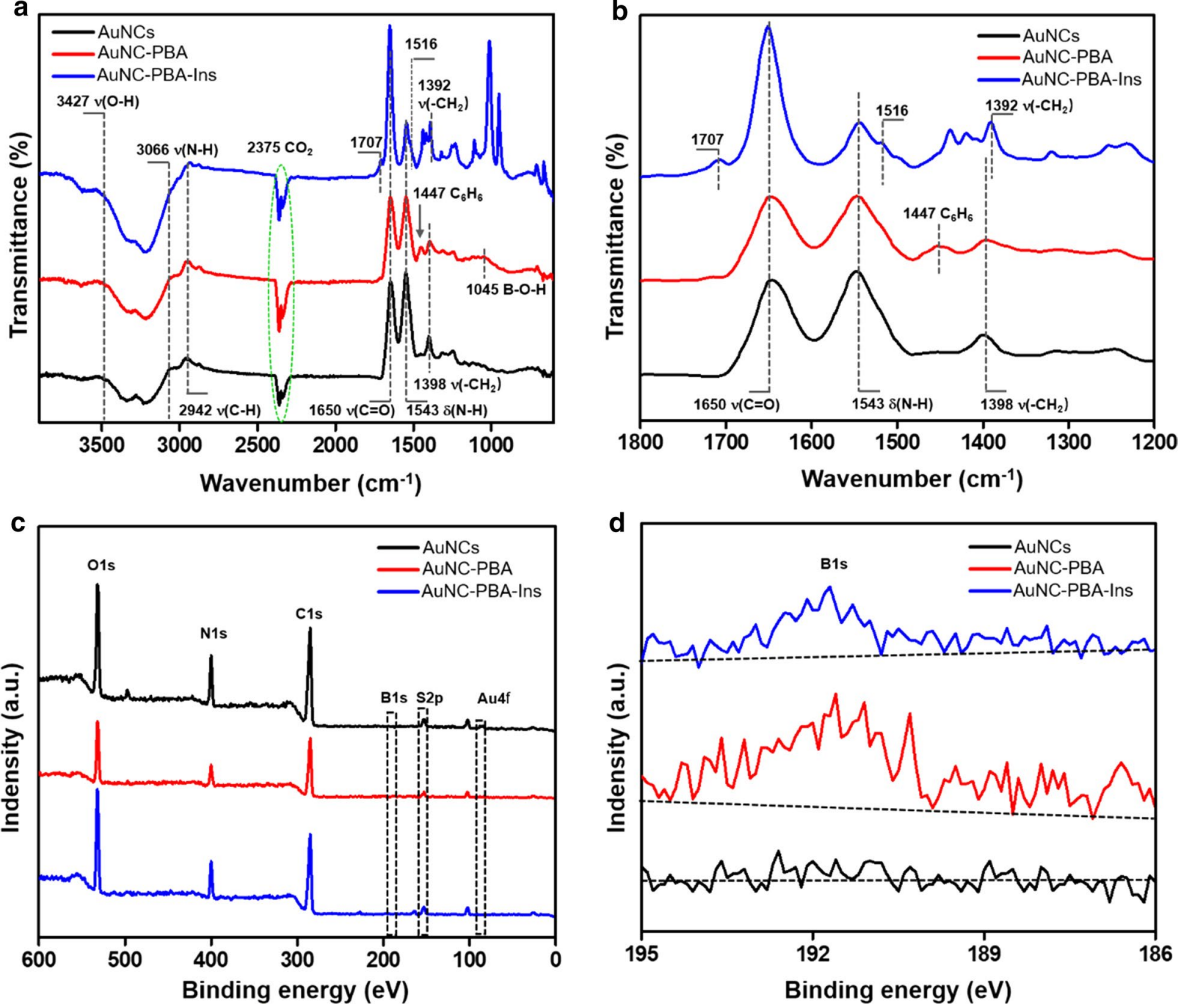
FTIR and XPS analysis of AuNCs. **a** FTIR wide spectra. **b** FTIR spectra at 1200–1800 cm^−1^. **c** XPS wide spectra. **d** High-resolution XPS spectra of B1s

We evaluated the changes in chemical structure during synthesis using FTIR (Fig. 3a, b). In the spectrum of AuNCs, we observed the characteristic amide I band (ν(C=O)) at 1650 cm^−1^ corresponding to BSA protein, which had a high proportion of α-helix [32], a strong band corresponding to primary amine scissoring (δ(N–H)) at 1543 cm^−1^ [32], –CH_2_ vibration at 1398 cm^−1^, a wide absorption band composed of the stretching vibration of the O–H bond at 3427 cm^−1^, primary amine ν(N–H) stretching mode at 3066 cm^−1^, and ν(C–H) stretching at 2942 cm^−1^ [32, 33] (Fig. 3a, b). In the spectrum of AuNC-PBA, we observed bonds involved in the benzene ring at 1447 cm^−1^ and B-O-H deformation at 1045 cm^−1^ (Fig. 3a, b). These are characteristic peaks from the PBA molecule [34, 35] and indicated the successful deposition of PBA on the AuNCs. In the spectrum of the AuNC-PBA-Ins complex, we observed a wide shoulder in the amide I band (1707–1650 cm^−1^) and a remarkable shift of the amide II band from 1543 to 1516 cm^−1^ (Fig. 3a, b). These are characteristics of insulin [36, 37] and revealed the successful combination of insulin on the complex.

We monitored the elemental changes during synthesis with XPS (Fig. 3c, d). On AuNC-PBA, the surface Au content slightly decreased compared to that of AuNCs (Table 2). In addition, AuNC-PBA exhibited substantial surface B atom (1.94%) (Table 2, Fig. 3c, d), due to the presence of PBA molecules. These results indicated the successful binding of PBA to AuNCs. On AuNC-PBA-Ins, because of the presence of insulin on the surface, the surface C and N contents were slightly increased, whereas the surface B content was slightly decreased compared to those on the AuNC-PBA (Table 2, Fig. 3d).

**Table 2:** Elemental compositions in samples assessed by XPS

Samples	Au (%)	S (%)	C (%)	N (%)	O (%)	B (%)
AuNCs	0.19	0.53	58.21	7.58	30.93	ND
AuNC-PBA	0.17	0.68	57.31	7.29	29.79	1.94
AuNC-PBA-Ins	0.14	0.57	65.95	8.79	21.21	1.02

*ND* not detected

We evaluated the loading capacity of insulin on AuNC-PBA-Ins complex. The gold concentration in AuNC-PBA-Ins solution, measured by ICP-MS, was 615.30 mg/L, and the concentration of insulin loaded onto the AuNC-PBA-Ins, evaluated by Bradford protein assay, was 0.79 ± 0.13 mM (Table 3). Accordingly, the AuNC-PBA-Ins had an insulin loading capacity of 1297.5 ± 211.1 μmol of insulin per gram of AuNCs (Table 3). To our knowledge, the insulin loading capacity of the AuNCs is approximately 17–95 times higher than that of conventional nanomaterials reported to date (Additional file 1: Table S2) [17, 22, 23]. The AuNC-PBA-Ins complex exhibited substantially higher insulin loading capacity, most likely because of the ultra-small size and ultra-large specific surface areas of the AuNCs.

**Table 3:** Characterization of drug loading capacity of AuNC-PBA-Ins

Au concentration (mg/L)	615.30
Loaded insulin (mM)	0.79 ± 0.13
Insulin loading capacity (μmol insulin per g AuNCs)	1297.5 ± 211.1

### In vitro insulin release from AuNC-PBA-Ins

As previously demonstrated, phenylboronic acid forms much more stable cyclic esters with the adjacent diols of saccharides than with vicinal diols [38, 39], suggesting that the linkage between PBA and G-Insulin on the AuNC-PBA-Ins complex can be cleaved by the introduction of excess saccharides (such as glucose) (Fig. 1e). Therefore, the release of G-Insulin from the AuNC-PBA-Ins complex would be sensitive to high glucose concentration (Fig. 1e).

**Fig. 4:**
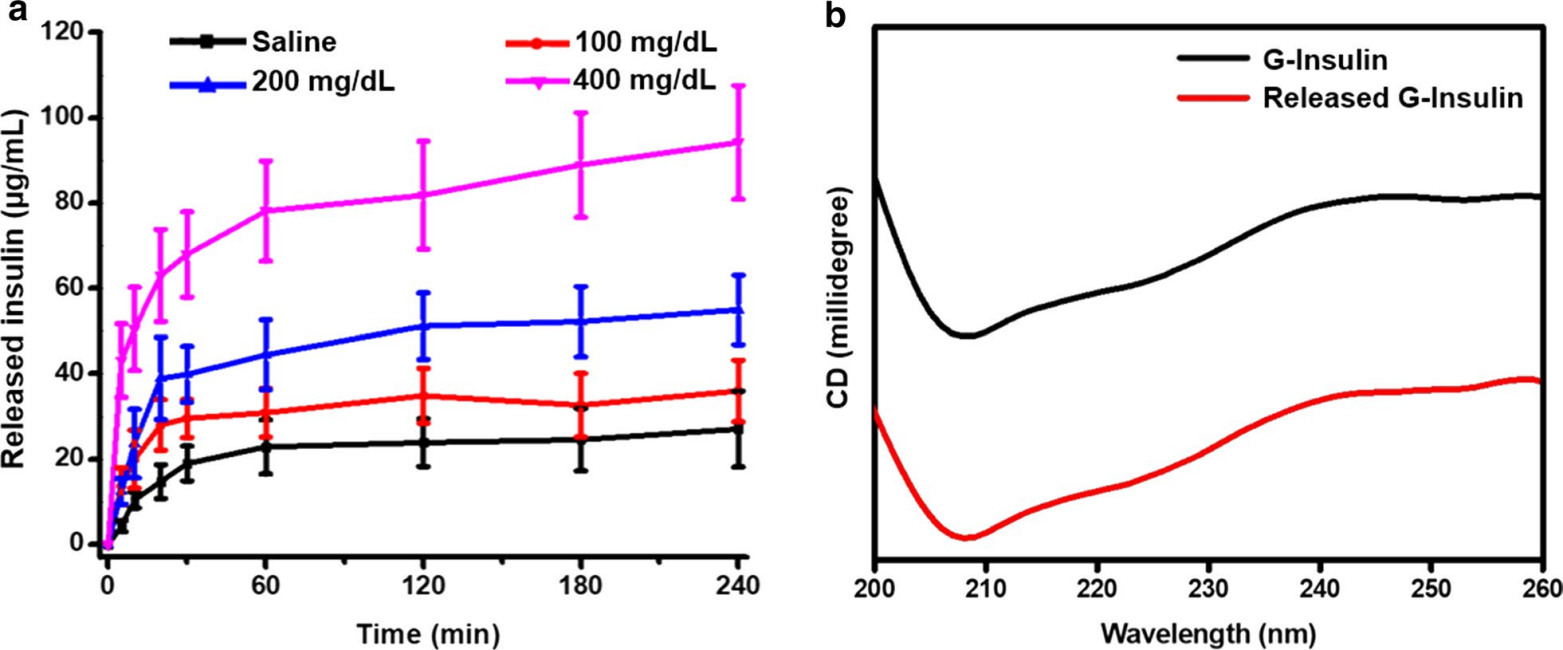
In vitro glucose-responsive insulin release from AuNC-PBA-Ins complex. **a** In vitro insulin release kinetics from the AuNC-PBA-Ins complex at different glucose concentrations at 37 °C. **b** CD spectra of insulin and insulin released from AuNC-PBA-Ins complex after incubation with 400 mg/dL glucose at 37 °C for 4 h

We evaluated the release of insulin from the AuNC-PBA-Ins complex at different glucose concentrations. At high glucose concentration (400 mg/dL), which simulated the hyperglycemic state in the body, the AuNC-PBA-Ins complex exhibited higher insulin release rate and more insulin release with incubation time (Fig. 4a). In the saline control or at lower glucose concentrations (100 mg/dL or 200 mg/dL), the AuNC-PBA-Ins complex displayed limited insulin release over time (Fig. 4a). Moreover, we found that the released G-Insulin from the AuNC-PBA-Ins complex maintained the conformational structure from G-Insulin by CD spectroscopy (Fig. 4b).

At high glucose concentration (400 mg/dL), the excess glucose competed with G-Insulin for the *cis*-diol binding site on PBA, thus the release of G-Insulin from the AuNC-PBA-Ins complex was activated (Fig. 1e). In contrast, at lower glucose concentrations (100 mg/dL or 200 mg/dL), the competition of glucose with G-Insulin for the *cis*-diol units on PBA was weakened and thus the release of G-Insulin from the AuNC-PBA-Ins complex was limited (Fig. 1e). The above results revealed that insulin release from the AuNC-PBA-Ins complex was a glucose-responsive process, and high glucose concentrations caused faster and higher insulin release from the nanocomplex (Fig. 4a). Acting as a smart insulin-releasing approach in the body, insulin release from the AuNC-PBA-Ins complex was facilitated at high glucose levels and can be employed to inhibit hyperglycemia in diabetes in vivo.

### Glucose regulation in type 1 diabetic mice

**Fig. 5:**
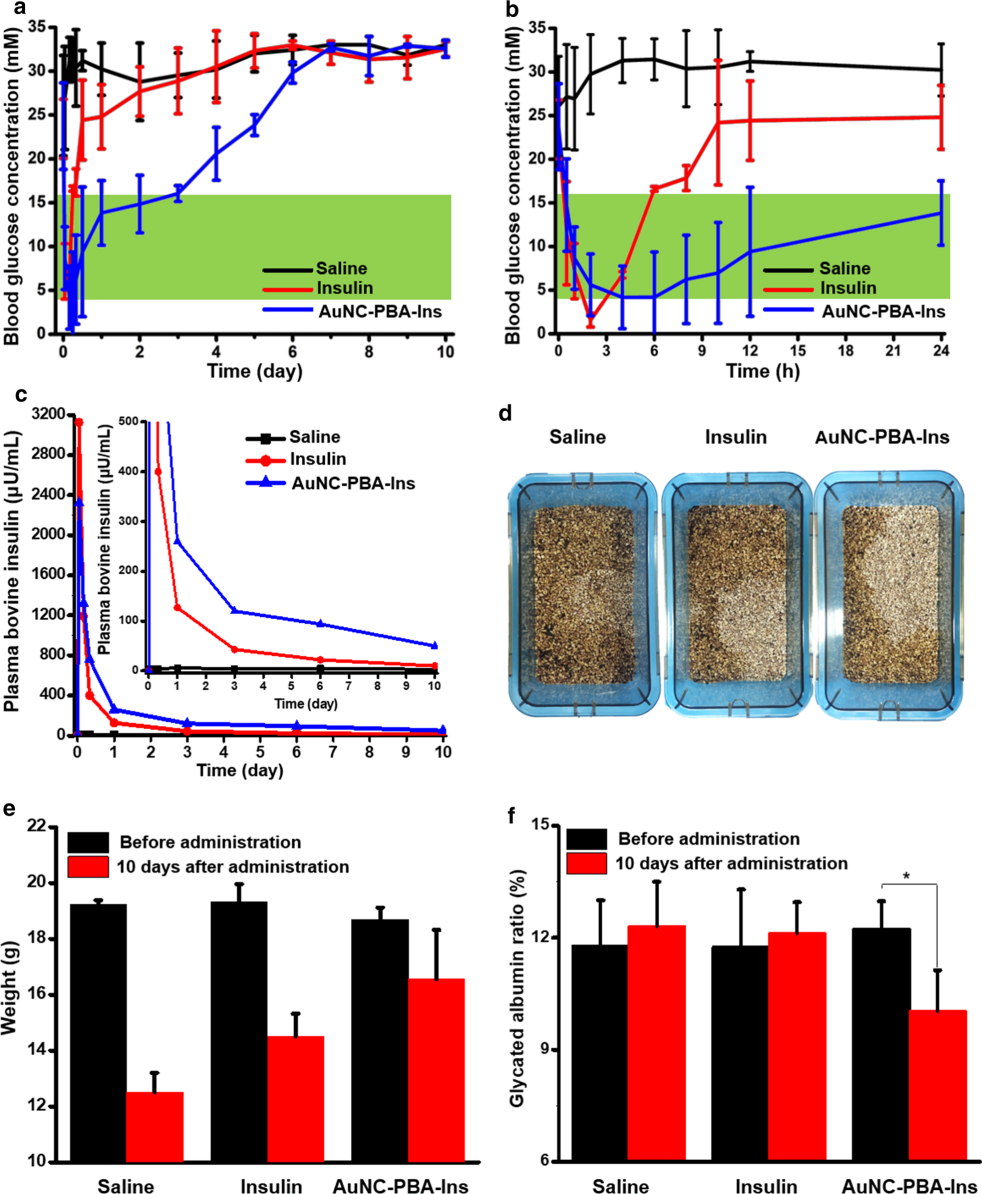
In vivo studies of the gold nanoclusters for type 1 diabetes treatment. **a**, **b** Blood glucose levels in type 1 diabetic mice after subcutaneous injection of various drugs. **c** Plasma bovine insulin concentration in diabetic mice after injection of various drugs. **d** Images of mouse cages 10 days after drug administration. **e** Mouse body weight before and 10 days after drug administration. **f** Percentage of glycated albumin in mice treated with different drugs. Mean ± S.D. (n = 5–6 per group). Significant difference was compared against the saline control, * 0.01 < p < 0.05; Student’s t-test

We examined the efficacy of the AuNC-PBA-Ins complex for glucose regulation in type 1 diabetes in vivo using STZ-induced type 1 diabetic mice (Additional file 1: Figure S4). In saline-treated mice, the blood glucose level remained in a hyperglycemic state over time (Fig. 5a). The administration of AuNC-PBA-Ins complex exhibited the most significant effects on blood glucose regulation (Fig. 5a). In mice administered with AuNC-PBA-Ins complex, the blood glucose level rapidly decreased to the normoglycemic range within 1 h after administration (Fig. 5b), and maintained in the normoglycemic range for up to 3 days without peaks of hyperglycemia or hypoglycemia (Fig. 5a, b). The blood glucose level then gradually raised to a hyperglycemic state (Fig. 5a). In contrast, in mice administered with pure insulin, the blood glucose level rapidly decreased to a hypoglycemic state 2 h after administration (Fig. 5b) and then rapidly increased to a hyperglycemic state 6 h after administration (Fig. 5a, b).

We detected the exogenous insulin in mice by bovine insulin ELISA. The plasma bovine insulin in mice administered with pure insulin was quickly cleared, resulting in a rapid decrease in plasma bovine insulin concentration 1 day after administration (Fig. 5c). However, plasma bovine insulin was detected in mice administered with AuNC-PBA-Ins complex over a longer range of time for about 3 days (Fig. 5c). Exogenous insulin in vivo allows the body to process glucose and avoid complications from hyperglycemia [2]. The average blood glucose levels of mice administered with AuNC-PBA-Ins complex increased because of the gradual decrease in insulin content over time (Fig. 5a, c).

Ten days after administration, the cage of mice treated with AuNC-PBA-Ins was drier with less urination than that of mice treated with saline or pure insulin (Fig. 5d, Additional file 1: Figure S5). The mice treated with AuNC-PBA-Ins complex had higher body weight compared to those treated with saline or pure insulin (Fig. 5e). The symptoms of diabetes are increased hunger, thirst, and urination and decreased body weight [40]. The body weight and condition of the cages indicated that the diabetic symptoms in mice treated with AuNC-PBA-Ins complex alleviated compared to those treated with saline or pure insulin (Fig. 5d, e). In addition, the higher body weight of mice treated with AuNC-PBA-Ins indirectly indicated no apparent toxicity from the AuNC-PBA-Ins complex (Fig. 5e).

We also evaluated the serum level of GA, a glycemic indicator in diabetic control [41], whereby diabetic patients aim to control the GA at low levels [41]. The GA level in mice treated with AuNC-PBA-Ins complex was decreased from 12.24 before drug administration to 10.04 at 10 days after drug administration, with a reduction of 17.9% 10 days after administration (Fig. 5f). In contrast, the administration of saline and pure insulin did not effectively decrease the GA level (Fig. 5f). The reduced GA level by AuNC-PBA-Ins indicated a better control of glucose in mice treated with the nanocomplex (Fig. 5f).

Taken together, in the in vitro glucose solution, the AuNC-PBA-Ins complex can responsively release insulin according to the environmental glucose concentration. In vivo, the administration of AuNC-PBA-Ins complex achieved better glycemic control in type 1 diabetic mice, and effectively maintained the blood glucose level in the normoglycemic range for up to 3 days.

## Conclusions

We have successfully developed a phenylboronic acid-functionalized gold nanocluster system (AuNC-PBA-Ins) for responsive insulin release and glucose control in type 1 diabetes. The AuNC-PBA-Ins complex released insulin rapidly in the hyperglycemic state, and effectively maintained the blood glucose level in the normoglycemic range for up to 3 days in type 1 diabetic mice. Our research constitutes a simple but effective method of intelligent insulin release and blood glucose regulation in diabetes. This gold nanocluster-based responsive insulin-releasing system mimics the function of blood glucose regulation of the pancreas in the body, which may have great applications in the theranostics in diabetes.

## Additional file

**Additional file 1: Figure S1.** Conversion of amino group into carboxyl group on the surface of AuNCs. (a) Amino group on AuNCs reacts with one aldehyde group on glutaraldehyde. (b) The other aldehyde group on glutaraldehyde reacts with the amino group on glycine. **Figure S2.** Preparation process of gluconic acid-modified bovine insulin (G-Insulin). **Figure S3.** Comparison of the bioactivity of pure insulin and gluconic acid-modified insulin (G-Insulin). (a) Normal mice were injected with pure insulin or G-Insulin. The blood glucose of mice was monitored for 90 min. (b) Mice without drug administration served as control, and the blood glucose of mice was recorded during the same period. **Figure S4.** The establishment of type 1 diabetic mouse model using streptozocin (STZ). (a) Change in glucose level during the induction of type 1 diabetic mice. The syringes indicate the days of STZ injection. (b) Images of mouse cages before STZ injection and after five injections of STZ. **Figure S5.** Change in percentage of wet area in mouse cages over time. **Table S1.** Characteristics of AuNCs evaluated by TEM and DLS. **Table S2.** Drug loading capacity of different nanocarriers.

## Data Availability

All data generated or analyzed during this study are included in this published article and its additional information files.
