# Supplementary material for: High drug-loading gold nanoclusters for responsive glucose control in type 1 diabetes
Source: J Nanobiotechnology. 2019 Jun 3;17:74. doi: 10.1186/s12951-019-0505-z (PMC6547569; doi:10.1186/s12951-019-0505-z)
Supplement: Supplementary file 1 — Additional file 1: Figure S1. Conversion of amino group into carboxyl group on the surface of AuNCs. (a) Amino group on AuNCs reacts with one aldehyde group on glutaraldehyde. (b) The other aldehyde group on glutaraldehyde reacts with the amino group on glycine. Figure S2. Preparation process of gluconic acid-modified bovine insulin (G-Insulin). Figure S3. Comparison of the bioactivity of pure insulin and gluconic acid-modified insulin (G-Insulin). (a) Normal mice were injected with pure insulin or G-Insulin. The blood glucose of mice was monitored for 90 min. (b) Mice without drug administration served as control, and the blood glucose of mice was recorded during the same period. Figure S4. The establishment of type 1 diabetic mouse model using streptozocin (STZ). (a) Change in glucose level during the induction of type 1 diabetic mice. The syringes indicate the days of STZ injection. (b) Images of mouse cages before STZ injection and after five injections of STZ. Figure S5. Change in percentage of wet area in mouse cages over time. Table S1. Characteristics of AuNCs evaluated by TEM and DLS. Table S2. Drug loading capacity of different nanocarriers. [file 12951_2019_505_MOESM1_ESM.docx]

**Additional e-file 1**

**High drug-loading gold nanoclusters for responsive glucose control in type 1 diabetes**

Yujie Zhang^1^, Mingxin Wu^1^, Wubin Dai^2^, Min Chen^3^, Zhaoyang Guo^1^, Xin Wang^1^, Di Tan^1^, Kui Shi, Longjian Xue^1^, Sheng Liu^1^, Yifeng Lei^1*^

^1^ School of Power and Mechanical Engineering & The Institute of Technological Sciences, Wuhan University, Wuhan 430072, China

^2^ School of Material Science and Engineering, Wuhan Institute of Technology, Wuhan 430205, China

^3^ Department of Internal Medicine & Geriatrics, Wuhan University Zhongnan Hospital, Wuhan 430071, China

* To whom correspondence should be addressed. Email: [yifenglei@whu.edu.cn](mailto:yifenglei@whu.edu.cn). School of Power and Mechanical Engineering & The Institute of Technological Sciences, Wuhan University, Wuhan 430072, China


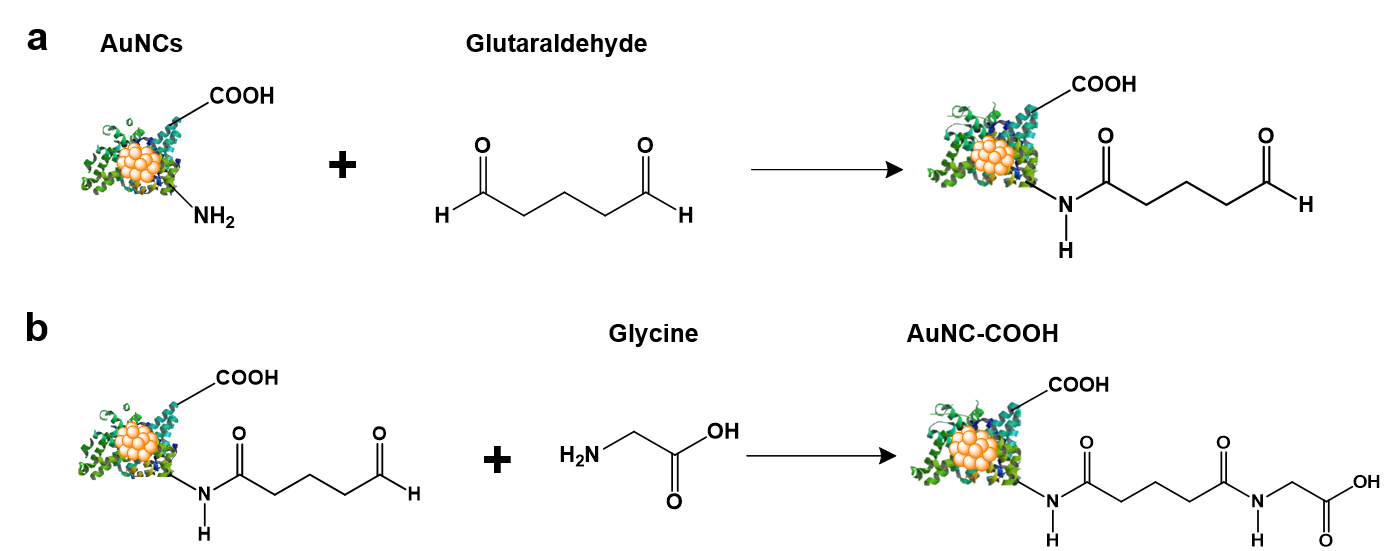


**Figure S1.** Conversion of amino group into carboxyl group on the surface of AuNCs. (**a**) Amino group on AuNCs reacts with one aldehyde group on glutaraldehyde. (**b**) The other aldehyde group on glutaraldehyde reacts with the amino group on glycine.


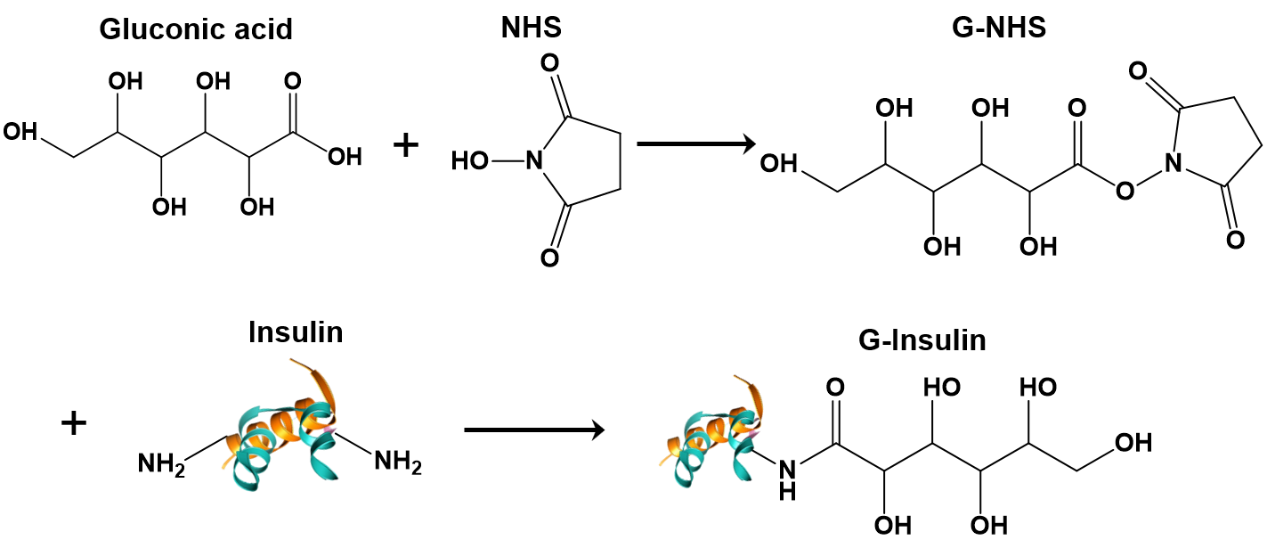


**Figure S2.** Preparation process of gluconic acid-modified bovine insulin (G-Insulin).


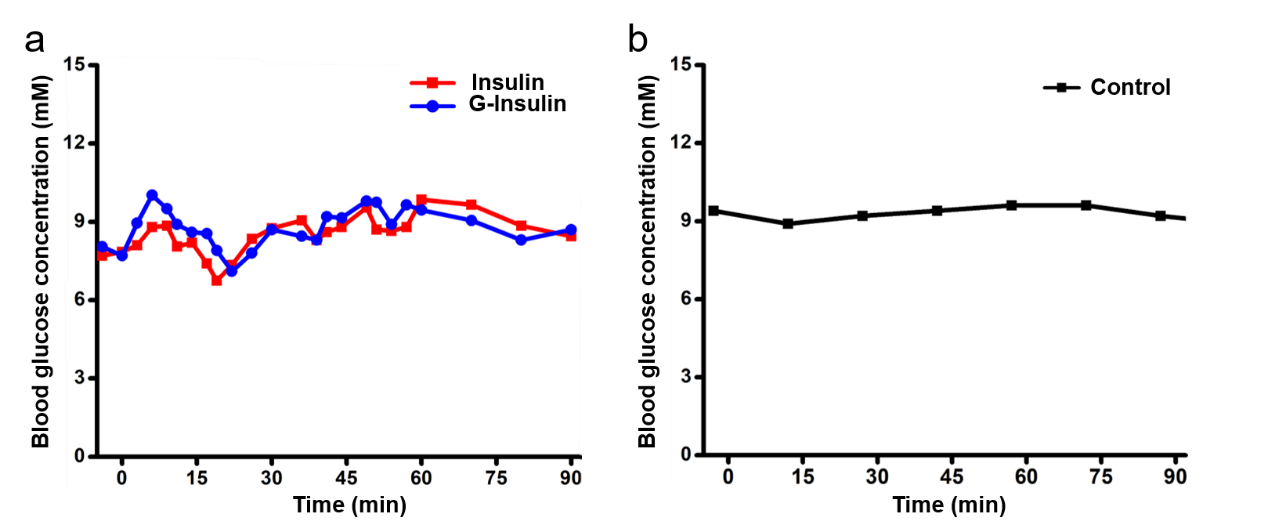


**Figure S3.** Comparison of the bioactivity of pure insulin and gluconic acid-modified insulin (G-Insulin). (**a**) Normal mice were injected with pure insulin or G-Insulin. The blood glucose of mice was monitored for 90 min. (**b**) Mice without drug administration served as control, and the blood glucose of mice was recorded during the same period.


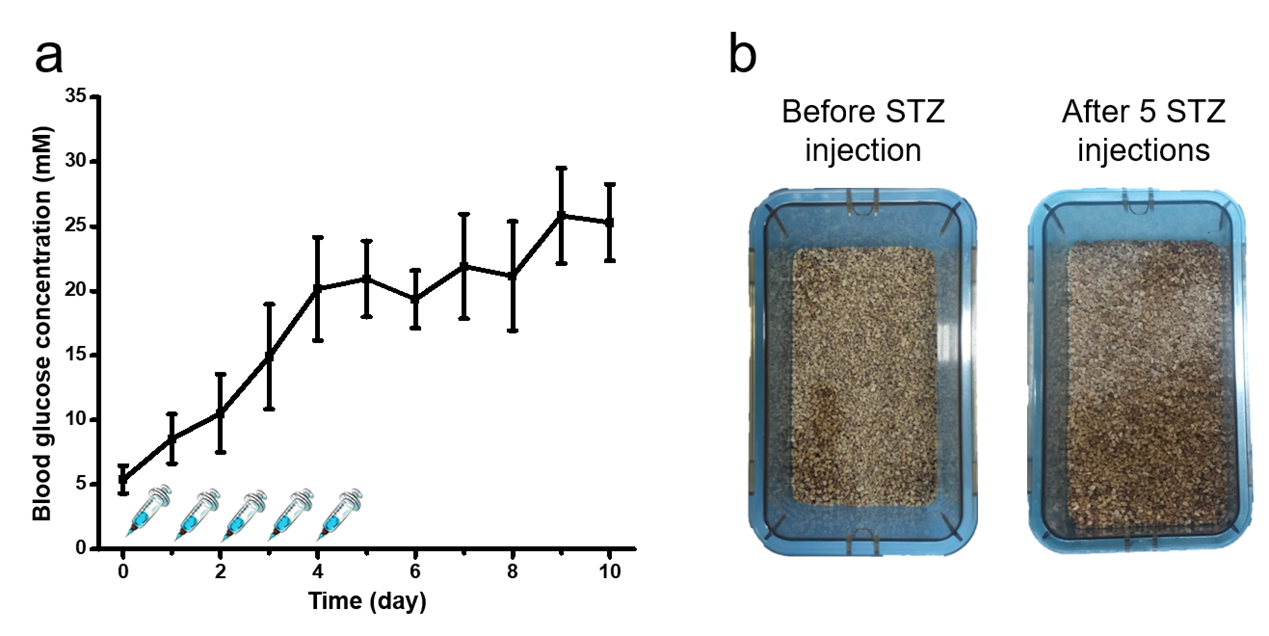


**Figure S4.** The establishment of type 1 diabetic mouse model using streptozocin (STZ). (**a**) Change in glucose level during the induction of type 1 diabetic mice. The syringes indicate the days of STZ injection. (**b**) Images of mouse cages before STZ injection and after five injections of STZ.


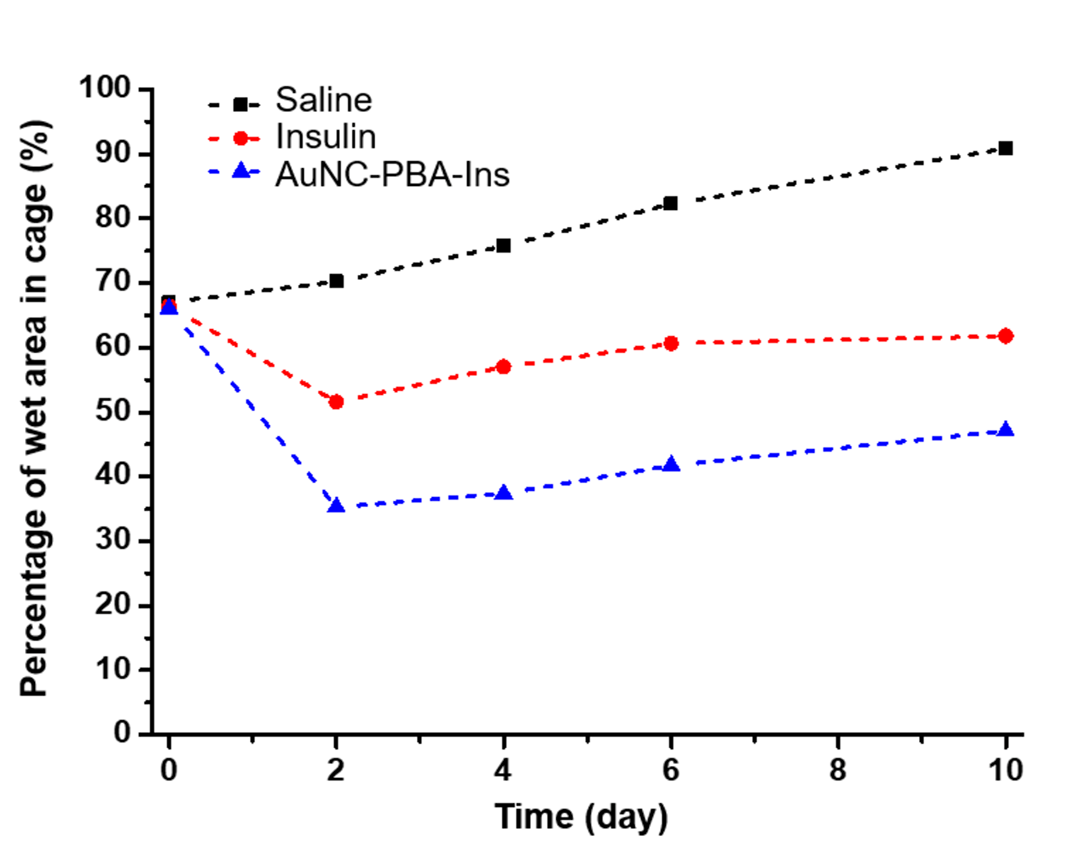


**Figure S5.** Change in percentage of wet area in mouse cages over time.

**Table S1. Characteristics of AuNCs evaluated by TEM and DLS**

|  | **Size by TEM**  **(nm)** | **Hydrodynamic size by DLS (nm)** | **Polydispersity**  **index (PDI)** | **Zeta potential**  **(mV)** |
| --- | --- | --- | --- | --- |
| AuNCs | 1.7 ± 0.2 | 5.4 ± 2.0 | 0.397 ± 0.104 | -34.2 ± 1.6 |
| AuNC-PBA | ND | 43.3 ± 5.2 | 0.421 ± 0.150 | -32.7 ± 1.4 |
| AuNC-PBA-Ins | 22.6 ± 2.0 | 142.4 ± 11.9 | 0.314 ± 0.065 | 18.2 ± 1.2 |

ND, not detected.

**Table S2. Drug loading capacity of different nanocarriers**

| **Nanocarriers** | **Loading capacity of insulin** | **Ref** |
| --- | --- | --- |
| AuNC-PBA-Ins | 1297.5 μmol insulin per g AuNCs |  |
| Mesoporous silica nanoparticles (MSNs) | 64 μmol insulin per g MSNs | [^1^](#_ENREF_1) |
| Nano-Networks | 7.9 wt % (13.6 μmol insulin per g chitosan-coated particles) | [^2^](#_ENREF_2) |
|  | 11.4 wt % (19.6 μmol insulin per g alginate-coated particles) | [^2^](#_ENREF_2) |
| Nanocapsules | 44.6 wt% (76.8 μmol insulin per g particles) | [^3^](#_ENREF_3) |

**Supplementary references**

1. Zhao, Y.; Trewyn, B. G.; Slowing, II; Lin, V. S., Mesoporous silica nanoparticle-based double drug delivery system for glucose-responsive controlled release of insulin and cyclic AMP. *J. Am. Chem. Soc.* **2009,** *131* (24), 8398-400.

2. Gu, Z.; Aimetti, A. A.; Wang, Q.; Dang, T. T.; Zhang, Y. L.; Veiseh, O.; Cheng, H.; Langer, R. S.; Anderson, D. G., Injectable nano-network for glucose-mediated insulin delivery. *ACS Nano* **2013,** *7* (5), 4194-4201.

3. Gu, Z.; Dang, T. T.; Ma, M.; Tang, B. C.; Cheng, H.; Jiang, S.; Dong, Y.; Zhang, Y.; Anderson, D. G., Glucose-responsive microgels integrated with enzyme nanocapsules for closed-loop insulin delivery. *ACS Nano* **2013,** *7* (8), 6758-66.
